# Preventive Effect of *Arctium lappa* Polysaccharides on Acute Lung Injury through Anti-Inflammatory and Antioxidant Activities

**DOI:** 10.3390/nu15234946

**Published:** 2023-11-29

**Authors:** Naiyan Lu, Jiayi Wei, Xuelei Gong, Xue Tang, Xuan Zhang, Wen Xiang, Samuel Liu, Cherry Luo, Xun Wang

**Affiliations:** 1 Department of Pulmonary and Critical Care Medicine, Jiangnan University Medical Center, Jiangnan University, Wuxi 214126, China; lunaiyan@jiangnan.edu.cn (N.L.); 2331320036@stmail.ntu.edu.cn (X.G.); 2 School of Food Science and Technology, Jiangnan University, Wuxi 214126, China; 6210113098@stu.jiangnan.edu.cn (J.W.); tangxue@jiangnan.edu.cn (X.T.); 8202106022@jiangnan.edu.cn (X.Z.); 3 National Engineering Research Center for Functional Food, Jiangnan University, Wuxi 214126, China; 4 School of Medicine, Nankai University, Tianjin 300350, China; 1120200668@mail.nankai.edu.cn; 5 Shenzhen Buddy Technology Development Co., Ltd., Shenzhen 518000, China; 15276521385@163.com (S.L.); cdlt0234@163.com (C.L.)

**Keywords:** *Arctium lappa*, polysaccharide, lung injury, LPS, inflammation

## Abstract

The objective of this study was to investigate the preventive effects of polysaccharides extracted from the roots of *Arctium lappa* (ALP) against acute lung injury (ALI) models induced by lipopolysaccharide (LPS). The polysaccharides were extracted and characterized, and their anti-inflammatory and antioxidant capacities were assessed. The findings demonstrated that ALP could mitigate the infiltration of inflammatory cells and reduce alveolar collapse in LPS-induced ALI in mice. The expression levels of the *pro*-inflammatory factor TNF-α decreased, while the anti-inflammatory factor IL-10 increased. Furthermore, the administration of ALP improved the activities of lung antioxidant enzymes, including SOD, GSH, and CAT, and lowered MDA levels. These results suggest that ALP exhibits a preventive effect on ALI and has potential as an alternative treatment for lung injury.

## 1. Introduction

Polysaccharides, a common type of biopolymer in nature, result from the linkage of monosaccharide molecules through covalent bonds, forming long-chain structures [1]. Polysaccharides have gained widespread recognition for their diverse applications, particularly in the fields of food and biomedicine [2]. Plants are a primary source of these compounds, as they not only form a fundamental part of plant cell walls but also serve as essential active components in traditional Chinese medicine and herbal remedies for various health conditions [3]. In the domains of functional foods and medicine, polysaccharides—especially those obtained from plant sources—are highly valued for their general non-toxicity and limited adverse effects [4]. Many plant-derived polysaccharides have been demonstrated to possess significant pharmacological properties [5,6].

Burdock (*Arctium lappa*) is a medicinal and edible plant belonging to the Asteraceae family, commonly found in Asian regions [7,8]. In China, *Arctium lappa* is predominantly marketed as an export product to other countries. However, the limited demand in international markets often results in market constraints, leading to the substantial wastage of this resource. The challenge arises from the fact that the plant’s roots are deeply embedded in the ground [9], making the harvesting process time-consuming and labor-intensive. This, in turn, escalates production costs and labor requirements, consequently restricting the market potential for *Arctium lappa*. To enhance the value of *Arctium lappa*, recent research has focused on investigating the bioactive compounds activated within the plant’s roots [10]. Polysaccharides derived from *Arctium lappa* (ALP), one of its primary bioactive compounds, are abundant in the roots of *Arctium lappa* [9]. These polysaccharides exhibit a range of pharmacological effects, including anti-inflammatory, antioxidant, hypoglycemic, and immunomodulatory activities [11,12,13]. For instance, polysaccharides isolated from *Arctium lappa* roots have been shown to enhance peritoneal macrophage activity, bolster immune system functionality [12], increase antioxidant enzyme activity, and mitigate vascular inflammation. Consequently, these properties reduce the risk of hypercholesterolemia and prevent cardiovascular diseases [14]. ALP could reduce cellular inflammation by regulating the balance of *pro*-inflammatory and anti-inflammatory factors, thereby treating rodents with colitis induced by dextran sulfate sodium (DSS) [15]. ALP also ameliorated carbon tetrachloride (CCl4)-induced liver injury in mice by inhibiting antioxidant defense mechanisms and activating the anti-inflammatory activity of the TLR4/NF-κB pathway [16]. Remarkably, despite these diverse therapeutic attributes, no research has been reported on the utilization of ALP for the treatment of acute lung injury (ALI).

ALI is a common clinical syndrome that often results in acute respiratory failure [17], typically characterized by severe pulmonary edema and uncontrolled inflammation of the lungs [18]. The inflammatory response and pulmonary edema can rapidly deteriorate lung function, which may ultimately result in respiratory failure and multiple organ dysfunction syndrome [19,20]. Inflammation and oxidative stress are the primary triggers of ALI development [21]. Suppressing inflammation and oxidative stress is an effective measure to repair ALI. Several highly effective anti-inflammatory drugs are currently used for ALI, but they also have certain limitations [22]. For example, high doses of simvastatin have been shown to improve organ dysfunction in ALI by reducing vascular inflammation and leakage, while the long-term, high-dose application of simvastatin may increase the risk of myopathy and liver injury [23,24]. Considering the robust pharmacological activity properties exhibited by ALP, coupled with its minimal adverse effects, we postulated that ALP may potentially serve as an effective agent in the management of pulmonary injuries. In this study, ALP was extracted and characterized. Additionally, in vitro and in vivo LPS-induced lung injury models were established to investigate ALP’s protective and anti-inflammatory effects in ALI.

## 2. Materials and Methods

### 2.1. Chemicals and Reagents

Fresh burdock roots were purchased from Xuzhou (Jiangsu, China). The supplier of mannose, rhamnose, galacturonic acid, glucose, galactose, and arabinose was Macklin Biochemical Technology Co. (Shanghai, China). LPS was provided by Sigma Aldrich (Saint Louis, MO, USA) for reagent use. ELISA kits (Cologne, Germany) for IL-1β (Cat#: EHJ 30568m), IL-6 (Cat#: EHJ 95903m), TNF-α (Cat#: EHJ 45111m), and IL-10 (Cat#: EHJ 47391m) were obtained from Huijia Biotechnology (Xiamen, China). Primary antibodies against TNF-α (Cat#: ab307164) and IL-10 (Cat#: ab9969) and HRP-conjugated goat anti-rabbit IgG H&L (ab6721) were obtained from Abcam Co. (Cambridge, MA, USA). The BCA protein quantification kit, RIPA lysate kit, and DAPI were purchased from Biyuntian Company (Changsha, China). All other chemical reagents were of analytical grade and purchased from Sinopharm Chemical Reagent (Shanghai, China).

### 2.2. Extraction of ALP

The fresh Actium lappa roots were washed, dried, and crushed to obtain the dried *Arctium lappa* root powder. A certain amount of powder was weighed, mixed with ultrapure water at a liquid ratio of 1:20 (g/mL), and extracted at 70 °C for 90 min. The resulting extract was then centrifuged at 4000 r/min for 15 min, after which the filtrate was collected. This step was repeated, and the mixture was concentrated to one-third of its original volume by rotating evaporation at 60 °C. After that, the filtrates were precipitated at 4 °C overnight with four times the volume of ethanol (95%). The precipitate was then resuspended, and after deproteinization using the trichloroacetic acid method, purified polysaccharides were obtained, which were referred to as ALP.

### 2.3. The yield and Chemical Composition Analysis

We weighed the mass of ALP before and after extraction and calculated the ALP yield using Formula (1). Subsequently, we used the phenol–sulfuric acid technique to calculate the total sugar content of ALP. The total sugar content was determined by creating a standard curve, with glucose as the standard and distilled water as a blank control. With bovine serum albumin (BSA) as the standard, quantitative analysis of protein content was conducted using the BCA protein concentration assay kit.

(1) $$\mathrm{Yield}=\frac{m_{1}}{m_{2}}\times 100\%$$

*m*_1_: The mass of ALP after isolation and extraction (g); *m*_2_: The raw material quality (g).

### 2.4. FT-IR and UV–Vis Analysis

The samples were analyzed by Fourier transform infrared spectroscopy (FT-IR). First, 5 mg of the sample was mixed with 500 mg of KBr powder, ground, and pressed into a tablet for measurement in the wavenumber range of 4000–500 cm^−1^. The ultraviolet–visible (UV-vis) spectral analysis of ALP was conducted in the UV range with a spectral range between 190 and 400 nm.

### 2.5. Molecular Weight and Monosaccharide Composition Analysis

The molecular weight of ALP was calculated using high-performance liquid chromatography (HPLC) after it was produced as a 5 mg/mL solution. HPLC was carried out using a Waters 2410 oscillometric refractive detector and an Empower workstation. The TSKgel G5000PWXL (300 mm × 7.8 mm) was employed. The mobile phase was 0.1 M sodium nitrate at a flow rate of 0.5 mL/min and an injection volume of 10 µL. Glucans with molecular weights of 2700, 9750, 135,030, 300,600, and 2,000,000 Da were utilized as standard references to construct a calibration curve to determine the molecular weight of ALP.

The monosaccharide composition analysis of ALP was determined according to a previous study [25]. One specific method utilized for this analysis involved the application of the 1-phenyl-3-methyl-5-pyrazolone (PMP) derivatization method. Initially, a 5 mg/mL solution of ALP was prepared and subsequently subjected to hydrolysis. A mixed standard solution was also prepared, which included mannose (Man), rhamnose (Rha), glucose (Glc), galactose (Gal), glucuronic acid (GlcUA), galacturonic acid (GalUA), and arabinose (Ara) at a concentration of 0.5 mg/mL. This standard solution was further diluted to create solutions with concentrations of 0.2, 0.1, 0.05, 0.02, and 0.01 mg/mL. In the next step, various concentrations of either the mixed standard solution or the ALP solution post-hydrolysis (200 µL) were thoroughly mixed with 0.3 M NaOH (100 µL). Subsequently, a 0.5 M PMP methanol solution was added, and the mixture was thoroughly agitated. The combined solution was then subjected to heating in a 70 °C water bath for 40 min, followed by cooling and the addition of 0.3 M HCl (100 µL) for neutralization. The next stage involved the addition of chloroform (400 µL), followed by 1 min of vortex mixing and centrifugation at 5000 rpm for 5 min, after which the lower organic layer was discarded. This procedure was repeated three times. Finally, the supernatant was collected, adjusted to a final volume of 1 mL, and subsequently analyzed by HPLC utilizing a 0.22 µm filter membrane.

Thermo Fisher Ultimate 3000 and a Thermo Fisher DAD detector were used to perform HPLC on a Galaksil-EF-C18 Bio column (250 mm × 4.6 mm × 5 mm; Galak, Wuxi, China). The HPLC method utilized a mobile phase consisting of 0.1 M phosphate-buffered saline (pH 6.8) as mobile phase A and acetonitrile as mobile phase B. The elution gradient was 83% A and 17% B. There was a flow rate of 1 mL/min, and UV absorbance was measured at 245 nm.

### 2.6. Microstructural and Surface Morphological Analyses

A cold-field-emission scanning electron microscope (cold-FE-SEM, Hitachi High-Tech Corporation, Tokyo, Japan, SU8100) with a resolution of 13 nm was used to examine the microstructure of the polysaccharides at an acceleration voltage of 3 kV. The polysaccharide samples were thinly spread on the sample stage, and a gold sputter coating was applied using a sputter coater. Subsequently, the cold-FE-SEM was utilized to capture and observe the microstructures of the samples. Images were taken at magnifications of 1000×, 2000×, and 5000×.

### 2.7. Evaluation of Cell Viability

The human lung epithelial cell line A549 was obtained from Procell Life Science & Technology Co., Ltd. (Wuhan, China). A549 cells were cultured in Kaighn’s modified Ham’s F-12K medium (Cat#: PM150910) containing 10% fetal bovine serum (FBS, Cat#: 164210-500) and 1% Penicillin–Streptomycin Solution (P/S, Cat#: PB180120) at 37 °C with 5% CO_2_. The medium was changed two to three times each week. Typically, cell passaging was performed when the cells reached 80–90% confluency. The third-passage cells were used for subsequent experiments. 

The impact of ALP on the viability of A549 cells was evaluated using the CCK-8 method. A549 cells were cultured for 24 h in 96-well dishes (2 × 10^3^ cells per well). Then, the polysaccharides were dissolved in a serum-free medium to different concentrations and filtered through a 0.22 μm membrane. LPS (10 μg/mL) was added to each well, followed by ALP treatment at different concentrations (100 μg/mL, 200 μg /mL, 400 μg/mL, 800 μg /mL), and incubated at 37 °C overnight in a humidified 5% CO_2_ atmosphere. The control group was cultured with an equal volume of serum-free medium. After that, cells were washed 2–3 times with phosphate-buffered saline (PBS). Subsequently, 200 μL of complete culture medium containing 20 μL of CCK-8 reagent was added to each well and further incubated at 37 °C for an hour. Finally, a microplate reader was used to detect the absorbance at 450 nm. 

### 2.8. Animals and Treatments

The rodents were all male C57BL/6J mice, about 8 weeks old, with a weight of 20 ± 2 g. The National Institutes of Health Guide for the Care and Use of Laboratory Animals was followed for conducting animal studies (NIH Publications No. 8023, revised 1978). The animals had ad libitum access to water and standard food, and they were fed in controlled settings with a temperature of 25 °C and a 12 h light/dark cycle. After one week of adaptive feeding, the animals were arbitrarily divided into four groups (*n* = 6 in each): control group, LPS group, ALP group, and LPS + ALP group. Mice in the ALP and LPS + ALP groups were orally administered ALP (400 mg/kg body weight/day) for 7 days. In both the control and LPS groups, equal volumes of saline were orally administered. On day 7, the LPS and LPS + ALP groups received a 5 mg/kg LPS intratracheal injection [26]. Pentobarbital was used to anesthetize the mice.

### 2.9. Histopathological Examination

Hematoxylin and eosin (H&E) staining of the lung tissues was conducted according to routine protocols. Firstly, the tissues were placed in embedding boxes and immersed in a 4% formaldehyde solution for overnight fixation. The next day, dehydration was carried out using a series of increasing concentrations of ethanol. Subsequently, the tissues were immersed in paraffin, and finally, 4 μm thick sections were cut using a microtome. After the sections were cut, they were air-dried and ready for subsequent experiments. Briefly, lung tissue sections were subjected to dewaxing and incubated in hematoxylin for 5 min and then stained with eosin. After dehydrating in a series of alcohol solutions, the sections were cleared in xylene. The pathological morphology of lung tissue was observed under a conventional inverted optical microscope, and five fields of view were randomly selected for measuring the alveolar cavity surface area.

### 2.10. The Determination of Oxidative Stress Indicators in Bronchoalveolar Lavage Fluid (BALF) and Serum

Using the appropriate kits and following the manufacturer’s instructions (Biosharp, Hefei, China), the concentrations of malondialdehyde (MDA), superoxide dismutase (SOD), catalase (CAT), and reduced glutathione (GSH) in the A549 cell lysates, BALF, and serum were assessed.

### 2.11. Evaluation of the Lung Wet/Dry Weight Ratio

After the cervical vertebrae were dislocated to euthanize the mice, blood was collected, and the right lungs of the mice were retrieved and weighed to determine their “wet weight”. The right lungs were then dried for 72 h in an oven set to 80 °C before being weighed once more to determine their “dry weight”. A measure of pulmonary edema was the wet/dry (W/D) weight ratio of the lungs.

### 2.12. Collection of BALF and Total Cell Count

Three alveolar lavages with 0.6 mL of PBS per mouse were used to obtain BALF. The total protein concentration in the BALF was determined using the BCA protein assay kit, and cytokines in the BALF were quantified. 

### 2.13. Measurement of Cytokine Levels in BALF and Serum

According to the manufacturer’s suggestions, enzyme-linked immunosorbent assay (ELISA) assay kits were used to measure the levels of IL-1β, IL-6, TNF-α, and IL-10 in the A549 cell supernatant, BALF, and serum. Initially, each ELISA plate was loaded with varying concentrations of standard samples and test samples, followed by incubation at 37 °C for 30 min. Subsequently, the plate was subjected to five washes using a *pre*-prepared washing solution. The enzyme-labeling reagent was then introduced, and further incubation was carried out at room temperature for an additional 30 min. After another round of washing, the color-developing solution was applied, and the reaction was halted. Finally, the absorbance was measured at a wavelength of 450 nm.

### 2.14. Immunohistochemical Examination

The paraffin-embedded lung tissue sections were deparaffinized and rehydrated with xylene and gradient ethanol. Endogenous peroxidase was blocked for 15 min with 3% H_2_O_2_, followed by antigen retrieval using high pressure. Following an hour-long blocking step with 10% goat serum, the sections were incubated with the anti-TNF-α antibody (Abcam, 1:250) and anti-IL-10 antibody (Abcam, 1:100) overnight at 4 °C. Then, the sections were incubated with HRP-conjugated goat anti-rabbit IgG H&L (1:200) at room temperature for an hour and visualized with DAB staining. Lastly, hematoxylin was used as a counterstain for cell nuclei.

### 2.15. Statistical Analysis

All data are expressed as means ± standard deviations (SDs). Differences between groups were determined by performing a one-way analysis of variance (ANOVA) using GraphPad Prism 9.5.0 software. The Tukey test was used for post hoc testing of all experimental groups. Differences were considered significant when *p* <  0.05.

## 3. Results

### 3.1. Chemical Composition and Structural Characterization Analysis of ALP

**Figure 1:**
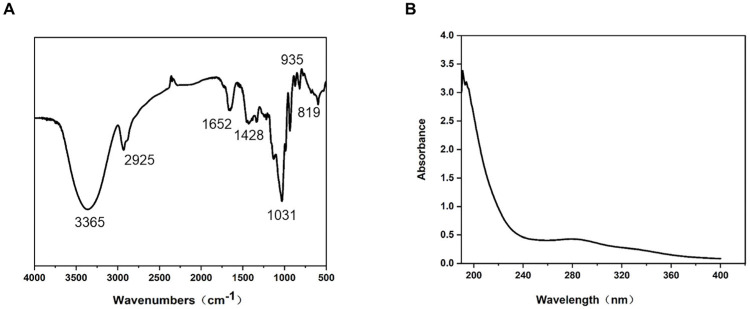
FT-IR and UV-vis analysis of polysaccharides from *Arctium lappa*. (**A**) Characteristic FT-IR spectrum of ALP. (**B**) UV-Vis spectrum of ALP in the range of 190–400 nm.

In this study, hot water extraction was used to separate ALP, and its yield and chemical composition were determined. The yield of ALP obtained was 1.94%, with a purity of 91.51% and a protein content of 6.46%. Afterward, the structure of ALP was characterized using UV−Vis and FT−IR. In the FT−IR spectrum of ALP (Figure 1A), the broad absorption band at 3365 cm^−1^ was attributed to the stretching vibration of O−H [27,28]. The absorption peaks at 2925 cm^−1^ and 1652 cm^−1^ resulted from the stretching vibrations of the C−H bond and carboxylate ion, respectively. These peaks are typical characteristics of polysaccharides, indicating the polysaccharide characteristics of ALP. The absorption peak at 1031 cm^−1^ suggested the presence of a pyranose form [29], indicating the existence of pyranoside in ALP. Additionally, the peak at 819 cm^−1^ indicated the presence of β−configuration glycosidic bonds in ALP [11]. In the UV−Vis spectrum, there were no characteristic absorption bands for proteins and nucleic acids in the wavelength range of 260−280 nm (Figure 1B), proving the absence of proteins and nucleic acids in purified ALP.

**Figure 2:**
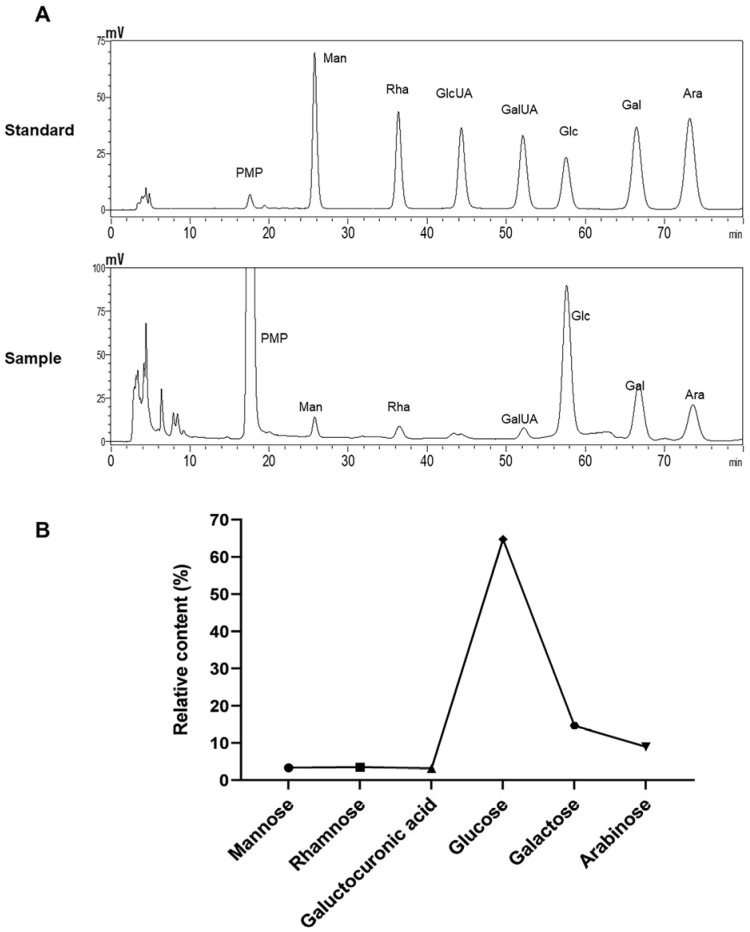
Monosaccharide composition of ALP. PMP derivatives of monosaccharide standard samples and polysaccharide hydrolysate from *Arctium lappa* chromatograms. (**A**) The upper graph depicts the chromatogram obtained from the standard sample, while the lower graph represents the chromatogram obtained from the sample. If peaks observed at the same time coordinates on both graphs, it indicates the presence of the same monosaccharide compound at that specific retention time. (**B**) Polysaccharide portion’s relative monosaccharide content.

Subsequently, the molecular weight and monosaccharide compositions of the ALP fractions were analyzed by high-performance liquid chromatography (HPLC), resulting in a molecular weight of 2340 Da. As shown in Figure 2, the results indicated that ALP was a heteropolysaccharide mainly composed of mannose, rhamnose, galacturonic acid, glucose, galactose, and arabinose in proportions of 3.321%, 3.497%, 3.195%, 64.725%, 14.638%, and 8.982%, respectively (Figure 2B). These findings are consistent with those of Zhang et al. [16,30].

**Figure 3:**
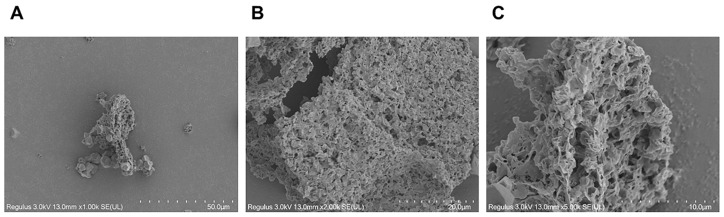
ALP was observed by SEM at magnifications of 1000 (**A**), 2000 (**B**), and 5000 (**C**).

Figure 3 shows the surface morphology and microstructure of ALP. ALP’s surface has some pebble-like granules mixed with a structure resembling a honeycomb. This construction appears to be rather dense, and there are multiple cavities visible on the surface.

### 3.2. ALP Improved Cell Viability, Oxidative Stress, and Inflammation

**Figure 4:**
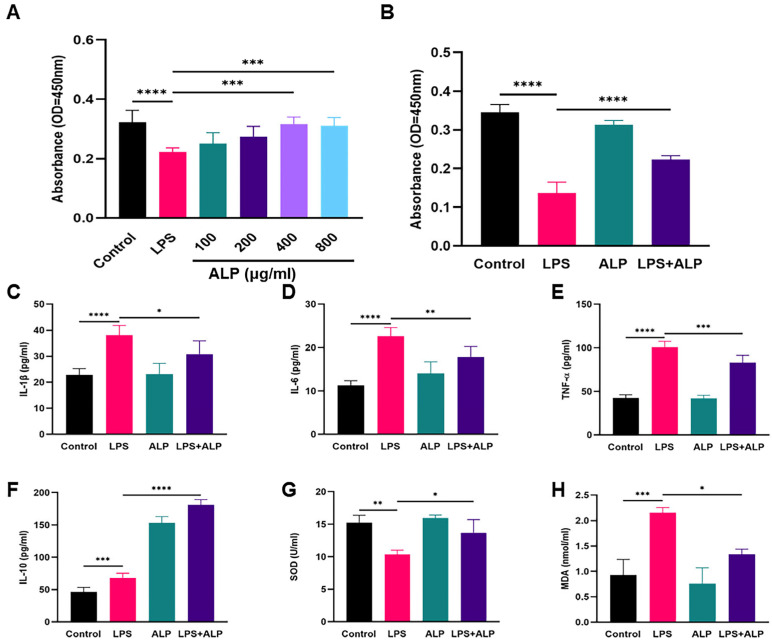
Impacts of ALP on LPS-induced A549 cell injury. (**A**) Cell viability measured by a CCK8 assay after treatment with different concentrations of ALP. (**B**) A549 cells after treatment with an ALP concentration of 400 μg/mL following LPS induction. (**C**–**F**) Effects of ALP on inflammatory cytokines TNF-α, IL-6, IL-β, and IL-10 in LPS-stimulated A549 cells. (**G**,**H**) Effects of ALP on MDA and SOD in LPS-stimulated A549 cells. * Indicates *p* < 0.05, ** indicates *p* < 0.01, *** indicates *p* < 0.0005, **** indicates *p* < 0.0001.

To determine the effect of ALP on A549 cell viability, a CCK-8 assay was performed [31]. As shown in Figure 4A,B, LPS significantly reduced cell viability. However, treatment with ALP reversed this trend. ALP at the 400 μg/mL concentration exhibited the best protective effect and was used for further experiments. Next, we explored ALP’s in vitro anti-inflammatory and antioxidant properties. The results suggested that the administration of ALP reduced the levels of IL-1β, IL-6, TNF-α, and MDA (Figure 4C–E,H) and increased the expression of IL-10 and SOD in LPS-induced A549 cells (Figure 4F,G).

### 3.3. ALP Improved the Pathological Morphology of LPS-Induced Lung Tissue in Mice

**Figure 5:**
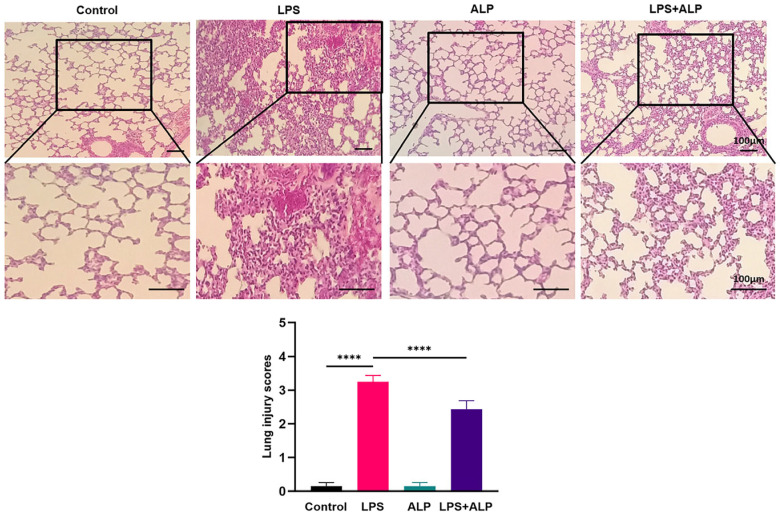
Effects of ALP on pathological alterations in mouse pulmonary tissue (×200, scale bar: 100 μm). **** Indicates *p* < 0.0001.

As depicted in Figure 5, LPS led to substantial damage to the alveolar structure, accompanied by noticeable inflammatory infiltration. The result of lung injury scoring demonstrated that the LPS group had significantly higher scores compared to the control group. However, the oral administration of ALP before exposure significantly reduced these scores. These findings emphasized the notable protective effects of ALP pretreatment, which effectively reduced alveolar wall thickening, prevented alveolar collapse, and resulted in lower hemorrhaging in the lung tissue.

### 3.4. Effect of ALP on Inflammation in LPS-Induced ALI Mice

**Figure 6:**
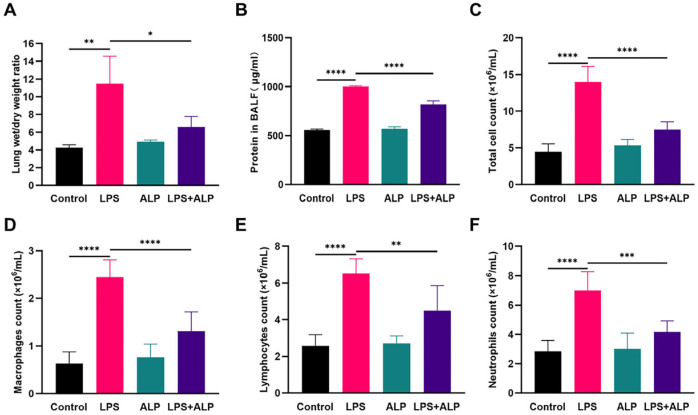
Effect of ALP on the lung wet/dry ratio (**A**), total protein levels in BALF (**B**), and inflammatory cell counts in the BALF of mice with LPS-induced ALI (**C**–**F**). * Indicates *p* < 0.05, ** indicates *p* < 0.01, *** indicates *p* < 0.0005, **** indicates *p* < 0.0001.

Figure 6A,B illustrate changes in the lung tissue W/D weight and the BALF protein content. Exposure to LPS led to a substantial increase in lung W/D weight and total protein, indicating the presence of pulmonary edema. However, the *pre*-administration of ALP effectively reduced pulmonary edema in ALI mice. Afterward, we counted the inflammatory cells 24 h after LPS injection. In comparison to the LPS group, the *pre*-administration of ALP resulted in a significant decrease in total cell counts, including macrophages, neutrophils, and lymphocytes (Figure 6C–F).

### 3.5. Effects of ALP on the Protein Expression of TNF-α and IL-10 in Mice

**Figure 7:**
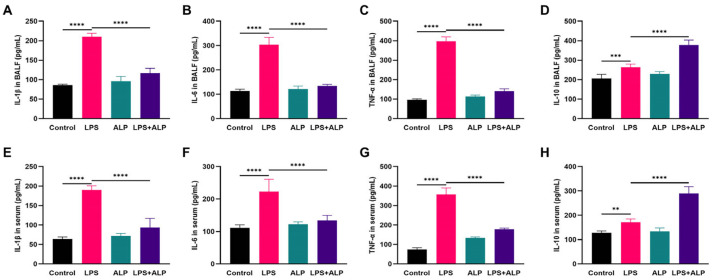
Effect of ALP on levels of inflammatory markers in LPS-induced ALI. (**A**–**D**) The levels of inflammatory marker variations in BALF (**E**–**H**) The levels of inflammatory marker variations in serum. ** Indicates *p* < 0.01, *** indicates *p* < 0.0005, **** indicates *p* < 0.0001.

**Figure 8:**
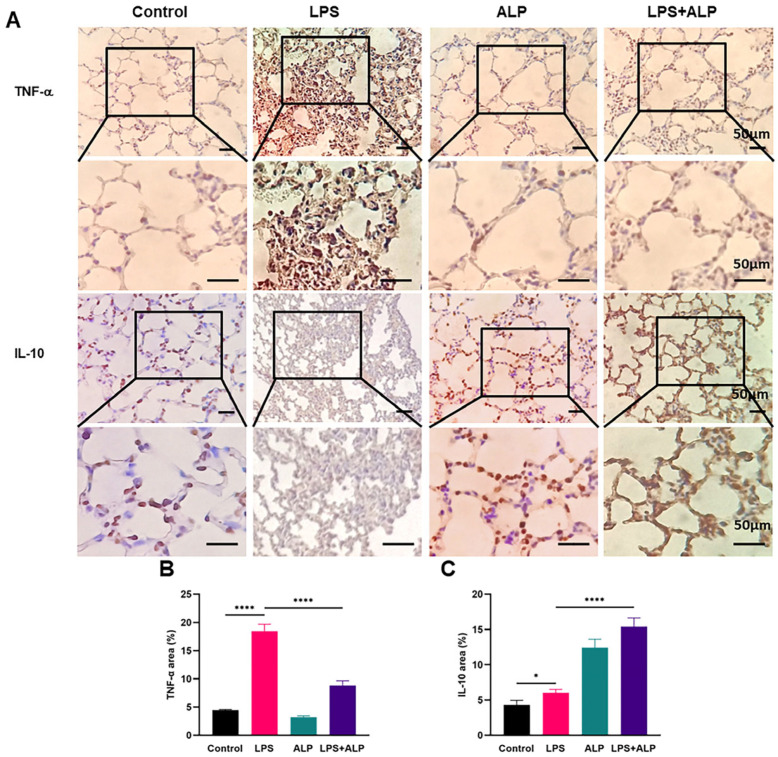
Effects of ALP on levels of TNF-α and IL-10 in lung tissues. (**A**–**C**) Representative images of TNF-α and IL-10 staining in lung tissues of mice and TNF-α- and IL-10-positive cells in lung tissues of mice (×400, scale bar: 50 μm). * Indicates *p* < 0.05, **** indicates *p* < 0.0001.

To further investigate the impact of ALP on LPS-induced lung inflammation, we used ELISA to measure the levels of inflammatory factors in the BALF and serum of mice. As seen in Figure 7, ALP administration dramatically enhanced the expression level of the anti-inflammatory factor IL-10 and decreased the release of the *pro*-inflammatory factors IL-1β, IL-6, and TNF-α in the BALF and serum. Furthermore, the anti-inflammatory effects of ALP were evaluated in lung tissues. As depicted in Figure 8, the LPS group displayed strong positive staining for TNF-α. Conversely, TNF-α staining was significantly reduced in the ALP prevention group, while IL-10 exhibited strong positive staining. These results indicate that ALP has the potential to mitigate ALI by balancing the levels of *pro*-inflammatory and anti-inflammatory factors.

### 3.6. Effect of ALP on Levels of Oxidative Stress Biomarkers in LPS-Induced ALI Mice

**Figure 9:**
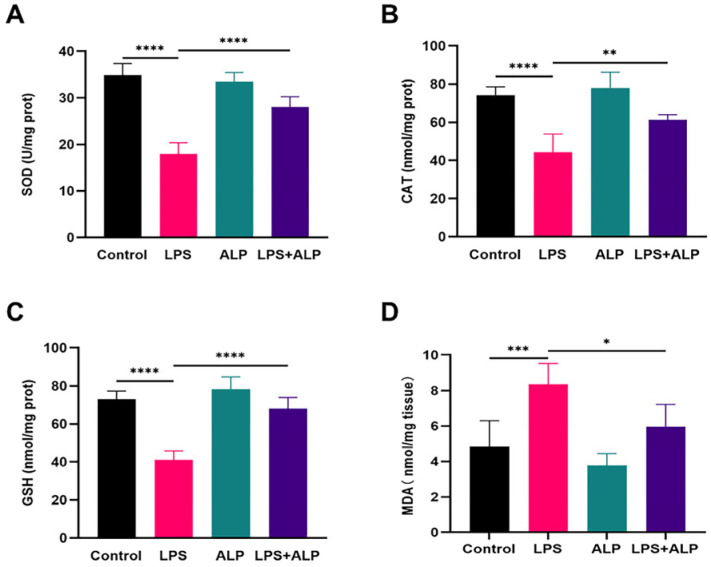
Impacts of ALP on antioxidant enzyme activity and lipid peroxidation markers in lung tissue. The activity of SOD (**A**) CAT (**B**) GSH (**C**) and MDA(**D**) in lung tissue. * Indicates *p* < 0.05, ** indicates *p* < 0.01, *** indicates *p* < 0.0005, **** indicates *p* < 0.0001.

The oxidative stress of lung injury was evaluated by measuring SOD, CAT, MDA, and GSH. Figure 9 demonstrates substantial decreases in SOD, CAT, and GSH activities in the LPS group of mice as compared to the control group, indicating that LPS injection interfered with the lung tissue’s antioxidant defense mechanism. However, when ALP was administered to ALI animals, the MDA levels dropped significantly, while the CAT, SOD, and GSH levels increased.

## 4. Discussion

ALI is a common and potentially life-threatening condition. Inflammation and oxidative stress are the primary mechanisms involved in its pathogenesis [32]. Currently, there are numerous drugs used in clinical practice to target inflammation and oxidative stress. For instance, drugs like methylprednisolone, dexamethasone, and prednisone belong to a widely used class of glucocorticoid medications [33]. They can reduce lung inflammation, improve respiratory function, and decrease tissue damage. However, the use of these drugs is also associated with a range of potential side effects, such as an increased risk of infections and a reduction in bone density [34]. Researchers have begun to concentrate on Chinese herbal constituents, notably polysaccharides, due to the minimal side effects and abundance of naturally occurring active chemicals present in traditional Chinese medicine. In recent years, ALP has gained widespread attention because of its beneficial anti-inflammatory properties and the advantage of having low toxicity or no toxicity. Previous studies have emphasized the anti-inflammatory and antioxidant properties of polysaccharides. For instance, Morchella esculenta polysaccharides can boost oxidative enzyme reactions and lower cell death, hence reducing oxidative stress [35]. Similar to this, *Codonopsis pilosula* polysaccharides [36] and *Lentinus edodes* polysaccharides [37] can prevent lung damage by inhibiting the production of *pro*-inflammatory cytokines, including TNF-α and IL-6. As a result, we speculated that ALP might be a useful medication for preventing ALI.

In this study, we extracted ALP from the roots of *Arctium lappa*. Our findings demonstrated that ALP reduced lung inflammation and decreased the mortality rate of A549 cells. Mice exposed to LPS exhibited structural damage in their lung tissue, primarily characterized by changes in the alveolar walls. The thickening of these walls indicated inflammation and impaired lung function. Alveoli, responsible for the exchange of oxygen and carbon dioxide during breathing, play a crucial role in this process [38]. When alveoli collapse, it signifies a severe impairment in the lung’s ability to efficiently exchange gases, resulting in breathing difficulties. In cases of ALI, hemorrhaging is a common and significant characteristic, further worsening respiratory problems. In contrast to the lung damage observed in the LPS-exposed group, the group that received *pre*-administered ALP displayed a protective effect. The most notable change was the reduction in thickened alveolar walls, indicating decreased inflammation and the prevention of alveolar collapse. Moreover, in mice that had received *pre*-administered ALP, there was no discernible bleeding from the lung tissue, suggesting that ALP preserved the integrity of blood vessels within the lung tissue.

Inflammation is one of the primary characteristics of ALI. One of the main characteristics of ALI is pulmonary edema, which reduces blood oxygenation and causes hypoxemia. The assessment of pulmonary edema is commonly based on alterations in the lung tissue W/D ratio and protein levels in the BALF [39]. When pulmonary edema occurs, increased capillary permeability may lead to the leakage of fluid and proteins into the alveoli and bronchi. Therefore, it is necessary to simultaneously measure the lung tissue W/D ratio and protein levels in the BALF to more fully assess the extent of pulmonary edema. In this work, we found that ALP could reduce pulmonary edema and lower the levels of inflammatory factors in the BALF. TNF-α and IL-10 are important cytokines that regulate the inflammatory response [40]. TNF-α is regarded as a major *pro*-inflammatory regulator with a variety of biological effects. Typically, it is significantly upregulated as ALI progresses [41]. IL-10 is an anti-inflammatory cytokine that mediates the process of countering *pro*-inflammatory immune responses, which is important in protecting host tissues from damage by *pro*-inflammatory cytokines [42]. The results further reflected that ALP could effectively reduce and mitigate inflammatory responses. Many studies have suggested that naturally sourced plant polysaccharides exhibit excellent anti-inflammatory properties. For instance, sulfated polysaccharides isolated from seaweed, after enzymatic digestion, demonstrate notable anti-inflammatory effects in RAW 264.7 cells stimulated with LPS [43]. In a mouse colitis model [15], the oral administration of ALP could alter the composition of the gut microbiota, significantly increasing the abundance of Firmicutes, Ruminococcaceae, Lachnospiraceae, and Lactobacillus while inhibiting the levels of Proteobacteria, Alcaligenaceae, Staphylococcus, and Bacteroidetes in the colitis mice. This suggests that oral ALP may alleviate inflammatory responses by regulating the abundance of various key microorganisms. Therefore, we speculated that ALP might influence the composition of the intestinal microbiota and alleviate inflammation in lung injury by affecting the breakdown of polysaccharides in the digestive tract.

Since the lungs are directly exposed to external environmental influences, they are susceptible to oxidative stress damage [44]. External factors such as pathogens and pollutants can trigger the generation of reactive oxygen species (ROS) [45], causing harm to lung tissues. ROS are a highly reactive group of oxidative molecules produced within cells [46]. When ROS are generated in excess, they can trigger lipid peroxidation, leading to the production of MDA [47]. This upsets the equilibrium between oxidation and antioxidation and thus lowers the activities of antioxidant enzymes like SOD and CAT. SOD and CAT represent two pivotal antioxidant enzymes [48]. Initially, SOD facilitates the elimination of superoxide anions, converting them into H_2_O_2_ [49]. Subsequently, CAT further catalyzes the decomposition of H_2_O_2_ into water and oxygen [49], ensuring the non-accumulation of H_2_O_2_ and thereby mitigating the deleterious effects of oxidative stress. Similarly, GSH is a crucial antioxidant that safeguards the body from oxidative damage by neutralizing free radicals [50]. Therefore, assessing the activities of SOD, CAT, GSH, and MDA is crucial for understanding the extent of lung damage. According to the results, LPS caused a decline in the activities of SOD, GSH, and CAT, along with an increase in MDA levels. However, these indices largely returned to normal levels with prior ALP intake. These results indicated that ALP could enhance antioxidant enzyme activity, aiding in the elimination of free radicals induced by oxidative stress, thus maintaining the redox balance [51]. The antioxidant capacity may be associated with the monosaccharide composition and ratio of ALP [52].

There are still several limitations in this research. On the one hand, as mentioned in previous research, A549 cells used in in vitro experiments cannot completely replace type II alveolar epithelial cells (ACEII) [53], even though they reflect the structure and function of alveoli to some extent. On the other hand, we also need to consider whether the LPS-induced lung injury model can fully simulate human lung injury, which requires further research and validation. Currently, ALP shows potential in the experimental treatment of ALI. However, future research and practical applications need to delve deeper into the treatment mechanisms of ALP and undergo more comprehensive clinical validation.

## 5. Conclusions

In summary, the anti-inflammatory and antioxidant properties of ALP have been validated in both in vivo and in vitro experiments. ALP improved the morphology of lung tissue, balanced *pro*- and anti-inflammatory factors, and reduced oxidative stress to prevent LPS-induced lung injury. Our findings imply that ALP could be a potential natural substitute for the treatment of lung damage due to its anti-inflammatory and antioxidant properties.

## Data Availability

All the data are available from the corresponding author upon reasonable request.
